# Bioequivalence, Food-Effect Assessment and Exploratory IVIVC of Two Extended-Release Tamsulosin 0.4 mg Formulations in Healthy Mexican Subjects

**DOI:** 10.3390/pharmaceutics18081008

**Published:** 2026-08-14

**Authors:** Omar Emmanuel Hernández Piña, Alberto Martínez Muñoz, Erika Gabriela Guido Ávila, Abraham Escobedo-Moratilla, Porfirio de la Cruz Cruz, José Trinidad Pérez-Urizar

**Affiliations:** 1 Asofarma de México S.A. de C.V., Av. Santa Fe 485, Lomas de Santa Fe, Contadero, Cuajimalpa de Morelos, Mexico City C.P. 05348, Mexico; ophernandez@adium.com.mx (O.E.H.P.); aescobedo@adium.com.mx (A.E.-M.); 2 Escuela Superior de Medicina, Instituto Politécnico Nacional, Salvador Díaz Mirón, Esq. Plan de San Luis S/N, Casco de Santo Tomás, Miguel Hidalgo, Mexico City C.P. 11340, Mexico; 3 Doctorado Institucional en Ingeniería y Ciencia de Materiales (DICIM), Universidad Autónoma de San Luis Potosí (UASLP), Av. Sierra Leona No. 550, Col. Lomas 2da. Sección, San Luis Potosí C.P. 78210, Mexico; 4 Facultad de Ciencias Químicas, Universidad Autónoma de San Luis Potosí, Av. Dr. Manuel Nava No. 6, Zona Universitaria, San Luis Potosí C.P. 78210, Mexico

**Keywords:** tamsulosin, benign prostatic hyperplasia, extended-release, bioequivalence, dose dumping, pharmacokinetics, in vitro–in vivo correlation

## Abstract

**Background/Objectives**: Tamsulosin extended-release (ER) formulations minimize peak-related vasodilatory adverse events in benign prostatic hyperplasia (BPH) treatment. This study assessed the pharmacokinetics and bioequivalence of a generic tamsulosin 0.4 mg ER formulation against the innovator under fasting and fed conditions. **Methods**: Two randomized, open-label, four-period crossover, single-dose trials were conducted in healthy Mexican males. Subjects received treatment following a 10 h fast or a high-fat meal, with a 7-day washout. Plasma tamsulosin was quantified over 72 h via LC-MS/MS. An exploratory in vitro–in vivo correlation (IVIVC) analysis was also performed. **Results**: Analyses included 32 (fasting) and 58 (fed) subjects. In both states, 90% confidence intervals for C_max_, AUC_0-t_, and AUC_0-∞_ geometric mean ratios fell entirely within the 80.00–125.00% bioequivalence limits. The formulations showed comparable dissolution (f_2_ = 92.47), with numerical deconvolution proving most predictive in exploratory IVIVC. Notably, the pharmacokinetic profile of the test formulation was consistent with maintained controlled drug release under both dietary conditions and showed no pharmacokinetic evidence of food-induced dose dumping. Both formulations were well-tolerated; all adverse events were mild, with no clinically significant orthostatic hypotension observed. **Conclusions**: The generic tamsulosin 0.4 mg ER formulation is bioequivalent to the reference product in both fasting and fed states. Its structural robustness prevents dose dumping, ensuring a favorable hemodynamic safety profile. Comparable dissolution and IVIVC findings further support their reliability in vivo performance and therapeutic interchangeability. ClinicalTrials.gov identifiers: NCT07698288 and NCT07698275.

## 1. Introduction

Benign Prostatic Hyperplasia (BPH) is one of the most common chronic conditions in aging men, characterized by the non-malignant proliferation of prostatic epithelial and stromal cells. This enlargement often leads to Lower Urinary Tract Symptoms (LUTS), including urinary frequency, urgency, nocturia, and poor or intermittent stream [1]. The prevalence of BPH is highly age-dependent, affecting approximately 50% of men by the age of 60 and rising to nearly 90% by age 85 [2]. If left untreated, progressive BPH can result in severe complications such as acute urinary retention, recurrent tract infections, and renal impairment, significantly deteriorating patients’ quality of life [3].

The pharmacological management of BPH-associated LUTS primarily relies on α_1_-adrenoceptor antagonists. Tamsulosin, a prominent member of this class, is widely prescribed due to its high receptor selectivity [4,5]. Unlike older, non-selective α1-blockers, tamsulosin exhibits substantially higher affinity for the α1A and α1D receptor subtypes, which are predominantly located in the smooth muscle of the prostate, bladder neck, and prostatic urethra. By inhibiting these receptors, tamsulosin effectively reduces smooth muscle tone, thereby alleviating dynamic bladder outlet obstruction and improving urine flow, with a minimal effect on the α1B receptors found in vascular smooth muscle [6].

Despite its uroselectivity, tamsulosin’s pharmacokinetic profile poses a significant clinical challenge. When administered as an immediate-release formulation, it results in rapid absorption and high peak plasma concentrations (C_max_), which can override its receptor selectivity and induce significant vasodilation [7]. This rapid systemic exposure is clinically associated with treatment-limiting adverse events, primarily orthostatic hypotension, dizziness, and syncope [7]. To mitigate these safety concerns, extended-release (ER) or oral controlled-absorption system (OCAS) formulations have been developed. These advanced delivery systems are designed to release the active pharmaceutical ingredient (API) continuously over 24 h, flattening the pharmacokinetic curve, reducing C_max_ fluctuations, and ensuring a steady therapeutic plasma concentration, thereby improving cardiovascular tolerability and patient adherence [8].

Pharmacokinetically, tamsulosin exhibits an absolute oral bioavailability of approximately 100% with a modified-release formulation; however, the rate and extent of systemic availability remain influenced by formulation-dependent release characteristics and gastrointestinal conditions. It is extensively bound to plasma proteins (approximately 99%, primarily to alpha1-acid glycoprotein) [9] and has a relatively small volume of distribution (approximately 16 L) [10]. Biotransformation occurs extensively in the liver via cytochrome P450 enzymes, particularly CYP3A4 and CYP2D6, so that less than 10% of the dose is excreted unchanged in the urine. The elimination half-life is approximately 10–15 h in healthy volunteers, making once-daily dosing suitable [11].

Crucially, the reliable performance of extended-release formulations depends heavily on gastrointestinal physiology, particularly the presence of food. Food intake significantly alters gastric emptying time, gastrointestinal transit, and splanchnic blood flow [12,13,14]. While many extended-release formulations utilize polymer matrix systems to prolong drug release, the risk of dose dumping is highly dependent on the specific formulation design and drug delivery technology. In matrix-based systems specifically, compromised structural integrity can lead to an unintended, rapid release of the active pharmaceutical ingredient, a phenomenon known as dose dumping, which may result in excessive systemic exposure and trigger severe hypotensive episodes with tamsulosin [15]. Conversely, food can slow the absorption rate, potentially leading to subtherapeutic levels. Therefore, demonstrating bioequivalence under both fasting and fed conditions is not merely a regulatory requirement but a critical scientific imperative to ensure that the generic ER formulation maintains its controlled-release integrity and prevents clinically relevant pharmacokinetic fluctuations regardless of the patient’s dietary state [16].

Although extended-release tamsulosin is widely used in clinical practice, pharmacokinetic data under fasting and fed conditions in Latin American populations, particularly among healthy Mexican subjects, remain limited. Generating such data is scientifically relevant given the reported interethnic variability in CYP3A4 and CYP2D6 activity, the principal enzymes involved in tamsulosin metabolism [17,18,19]. Therefore, the present study aimed to comprehensively characterize the pharmacokinetic profiles and unequivocally establish the bioequivalence of two tamsulosin 0.4 mg extended-release tablet formulations in healthy Mexican volunteers under both fasting and standardized fed conditions.

## 2. Results

### 2.1. In Vitro Dissolution Profiles

**Figure 1:**
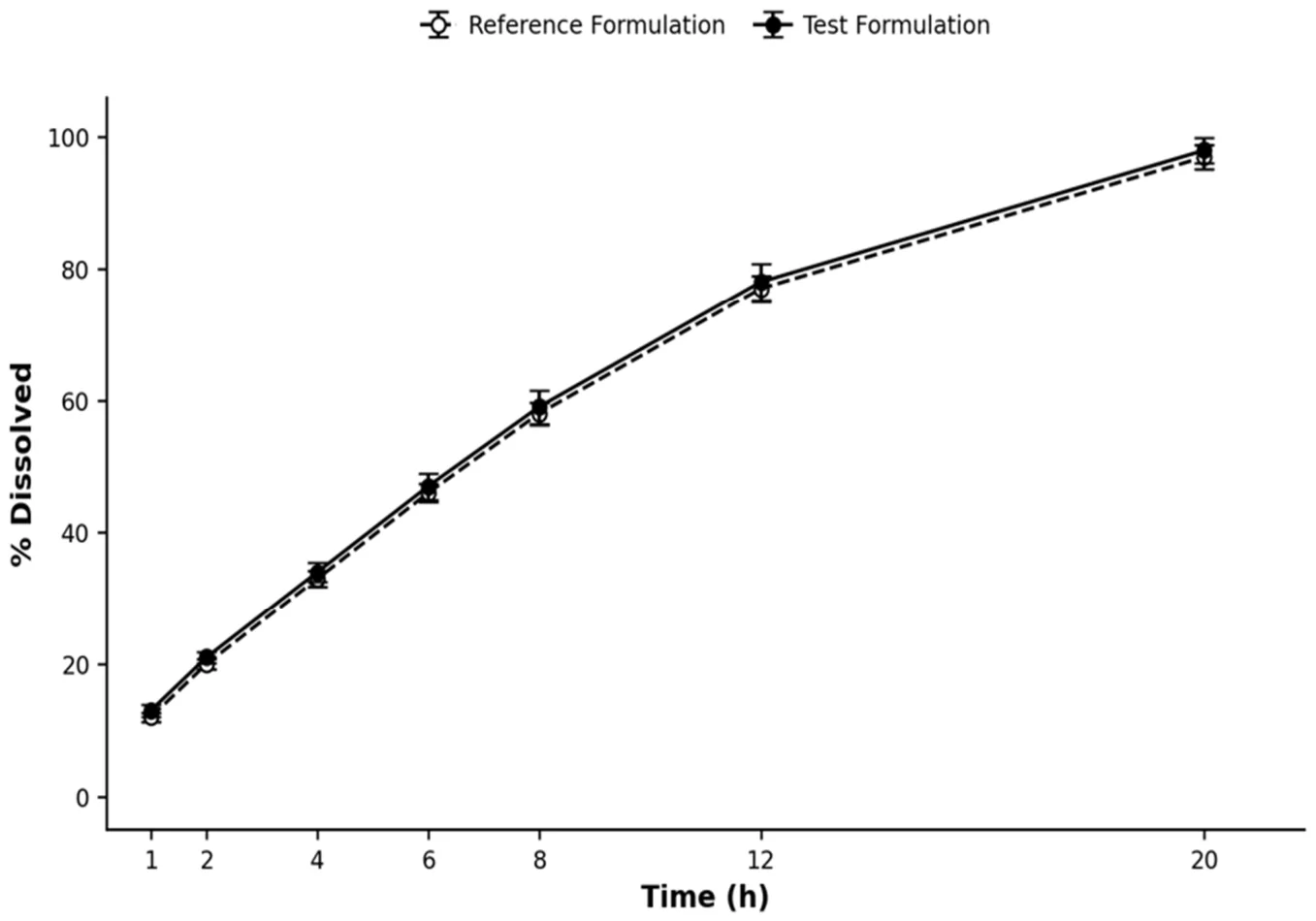
Mean in vitro dissolution profiles of the test (solid black line with filled circles) and reference (dashed black line with open circles) extended-release tamsulosin 0.4 mg formulations (n = 12). Data are expressed as the mean ± standard deviation (SD).

The mean dissolution profiles of the test and reference extended-release tamsulosin formulations are presented in Figure 1. Dissolution testing was performed under quality-control conditions using USP Apparatus II in phosphate buffer (pH 6.8). Both formulations exhibited gradual and controlled drug release across all sampling times, consistent with the intended extended-release characteristics of the dosage form. The dissolution profiles were highly similar, with a difference factor (f_1_) of 2.04 and a similarity factor (f_2_) of 92.47. These values satisfy the established acceptance criteria for profile equivalency (f_1_ ≤ 15 and f_2_ ≥ 50), indicating comparable in vitro release performance between the test and reference formulations.

### 2.2. Demographic Characteristics

The two independent bioequivalence trials enrolled a combined total of 100 healthy male subjects of Mexican descent, with 34 individuals allocated to the fasting protocol and 66 to the fed protocol. A comprehensive overview of the participants’ baseline demographic variables, including age, body weight, height, and body mass index (BMI), is provided in Table 1.

Pre-dose demographic evaluations revealed a high degree of homogeneity between the two study groups. Volunteers in the fasting study had a mean age of 36.8 ± 10.5 years and an average BMI of 23.6 ± 2.5 kg/m^2^. Comparable physical characteristics were observed in the fed study, presenting a mean age of 38.5 ± 9.7 years and a mean BMI of 24.5 ± 2.2 kg/m^2^. This physical uniformity ensures that the populations were adequately matched and comparable before drug administration.

**Figure 2:**
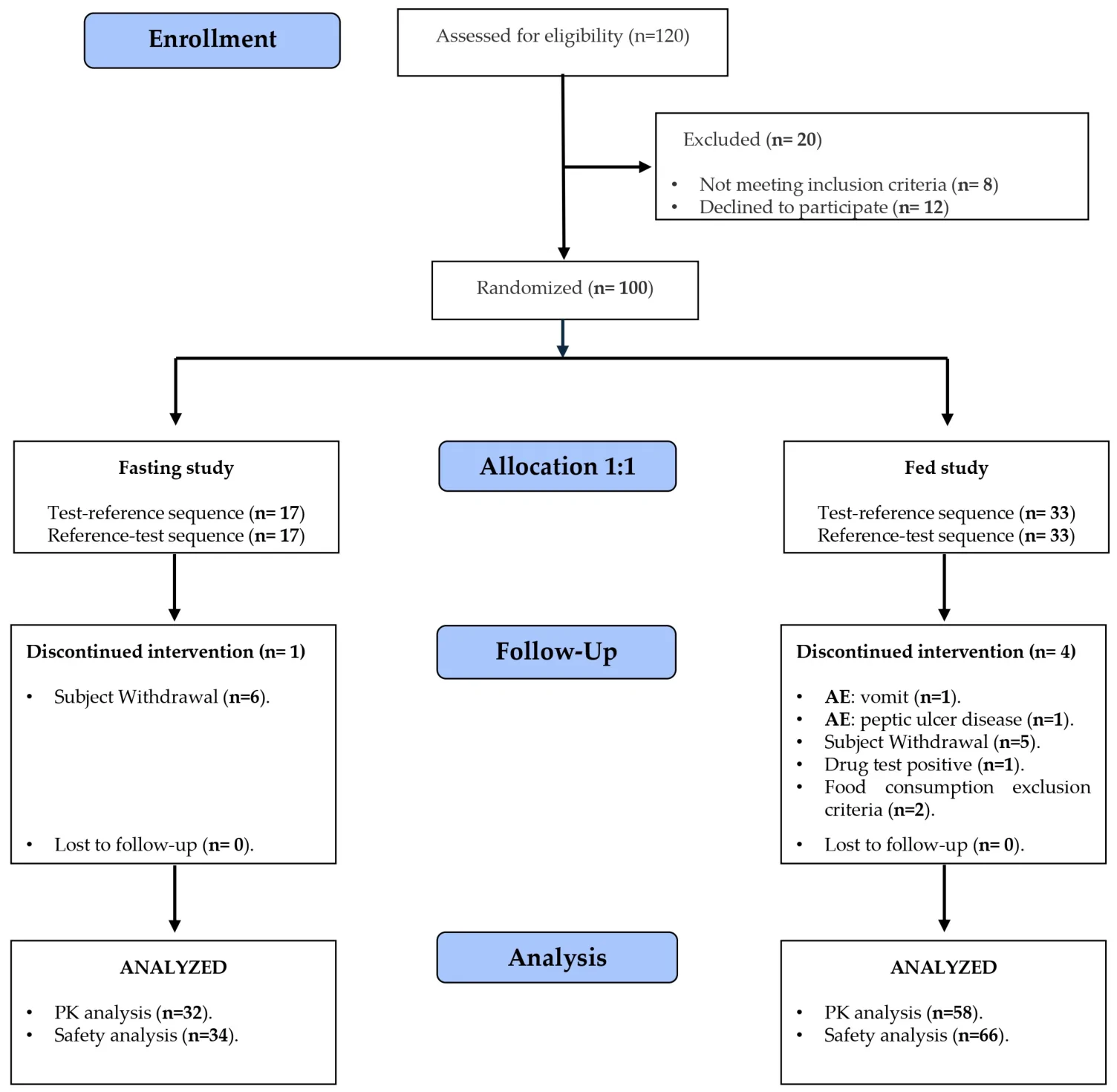
Study flowchart. n: number of subjects; PK: pharmacokinetic.

Throughout the clinical phase, specific participant withdrawals occurred; the exact details (screening, randomization, sequence allocation, and follow-up) are mapped in the consolidated CONSORT flow diagram (Figure 2). Ultimately, the clinical phase was successfully concluded by 28 participants in the fasting study and 56 participants in the fed study. In accordance with the predefined per-protocol (PP) criteria, subjects who completed at least one Test and one Reference period and provided evaluable pharmacokinetic data without major protocol deviations remained eligible for inclusion in the pharmacokinetic and bioequivalence analyses, regardless of study completion status.

### 2.3. Pharmacokinetics and Bioequivalence

**Figure 3:**
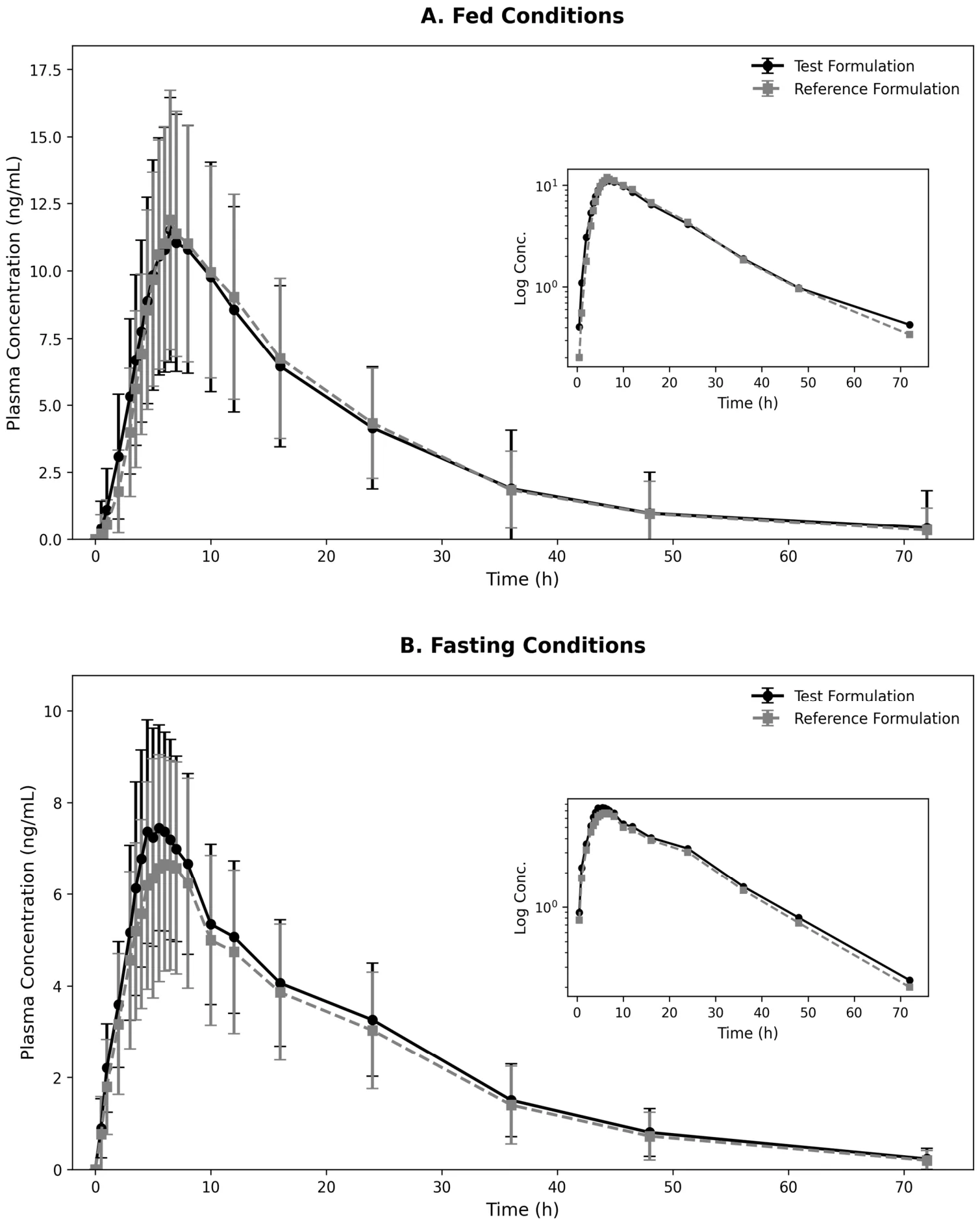
Mean plasma concentration–time profiles of tamsulosin following a single oral administration of the test (solid black line with circles) and reference (dashed gray line with squares) 0.4 mg extended-release tablets under fed (**A**) and fasting (**B**) conditions. Data are expressed as the mean ± standard deviation (SD). Insets show the corresponding semi-logarithmic profiles.

Following administration of the test and reference extended-release tamsulosin 0.4 mg formulations, the mean plasma concentration-time profiles closely mirrored each other in both the fasting and fed-state evaluations (Figure 3).

A comprehensive summary of the primary and secondary pharmacokinetic endpoints—including C_max_, t_max_, AUC_0-t_, AUC_0-∞_, and t_1/2_—is detailed in Table 2. In both dietary conditions, the absorption rate and overall systemic exposure were remarkably similar between the generic test product and the innovator reference drug.

Bioequivalence was statistically confirmed by calculating 90% confidence intervals (CIs) for the Test-to-Reference geometric mean ratios (GMRs) of the log-transformed primary parameters. As outlined in Table 3, all metrics met regulatory acceptance thresholds. Under fasting conditions, the 90% CIs were 103.33% to 120.32% for C_max_, 100.43% to 116.58% for AUC_0-t_, and 105.03% to 120.76% for AUC_0-∞_. Similarly, under fed conditions, the corresponding 90% CIs ranged from 89.49% to 100.04% for C_max_, 94.14% to 104.88% for AUC_0-t_, and 93.52% to 103.95% for AUC_0-∞_.

The within-subject coefficient of variation (CV_intra_) ranged from 17.4% to 26.2% across the evaluated PK parameters. Under fasting conditions, CV_intra_ values were 26.2%, 19.7%, and 17.4% for C_max_, AUC_0-t_, and AUC_0-∞_, respectively, whereas corresponding values under fed conditions were 22.3%, 24.6%, and 23.8%. These results indicate moderate intra-subject variability and are consistent with the narrow confidence intervals and high statistical power observed for all primary bioequivalence metrics.

Given that all 90% CIs for both independent studies are fully contained within the 80.00–125.00% bioequivalence acceptance margin, the therapeutic interchangeability of the test tamsulosin formulation is unequivocally supported regardless of the patient’s prandial state. Furthermore, Analysis of Variance (ANOVA) applied to the log-transformed data revealed no statistically significant effects (*p* > 0.05) regarding sequence, period, or treatment. This lack of significance confirms the validity of the two-way crossover design, appropriate washout periods, and the overall statistical robustness of the bioequivalence assessment.

### 2.4. Food-Effect Evaluation

To provide an exploratory descriptive assessment of the influence of food on the pharmacokinetic performance of the evaluated extended-release tamsulosin formulations, pharmacokinetic parameters obtained from the independent fasting and fed bioequivalence studies were compared. Fed-to-fasting (Fed/Fasting) ratios were calculated separately for the Test and Reference formulations using the arithmetic mean values of C_max_, AUC_0-t_, and AUC_0-∞_ (Table 4).

Food intake increased systemic exposure in both formulations. For the Test formulation, the Fed/Fasting ratios were 1.53, 1.45, and 1.55 for C_max_, AUC_0-t_, and AUC_0-∞_, respectively. Corresponding ratios for the Reference formulation were 1.79, 1.55, and 1.61, respectively. In both formulations, t_max_ remained prolonged under fed conditions, increasing from 5.56 to 6.67 h for the Test formulation and from 5.82 to 6.61 h for the Reference formulation. Overall, these findings indicate that food modified systemic exposure while maintaining the prolonged absorption profile characteristic of extended-release formulations.

### 2.5. Safety and Tolerability Analysis

In both clinical trials, the safety and tolerability of the test and reference extended-release tamsulosin 0.4 mg formulations were rigorously evaluated. Throughout the entire clinical program, no serious adverse events (SAEs) were reported, and all participants recovered completely without any long-term sequelae.

Overall, a notably higher incidence of adverse events (AEs) was observed during the fed study (32 events) than during the fasting study (8 events).

In the fasting study, a total of 8 AEs were documented, with 5 (63%) after administration of the test formulation and 3 (37%) after administration of the reference formulation. The reported events included dizziness, headache, abdominal pain, nasal congestion, and odynophagia. Notably, all 8 events in this study required pharmacological intervention to resolve symptoms.

In the fed study, 32 AEs were recorded, comprising 17 (53%) events associated with the test medication and 15 (47%) with the reference product. The safety profile in this prandial state primarily consisted of headache (18 events) and dizziness (5 events), followed by nausea, pharyngitis, vomiting, diarrhea, acid peptic disease, and dyspepsia. Among these, 19 (59.4%) events required pharmacological treatment.

A comprehensive breakdown of the frequency and distribution of all documented AEs across both dietary conditions is provided in Table 5.

As detailed in Table 5, the distribution and frequency of adverse events (AEs) were highly comparable between the test and reference treatment arms within each study. Although a higher overall incidence of AEs was observed in the fed study compared with the fasting study, no consistent pattern or clinically relevant differences were detected between the generic and innovator formulations. All documented events were consistent with the known safety and pharmacological profile of tamsulosin.

Given that the clinical program enrolled only male volunteers, demographic comparisons of AE frequency by gender were not applicable. Across both dietary conditions, the most reported adverse events were headache and dizziness, with gastrointestinal and upper respiratory events occurring less frequently.

Participant withdrawals during the clinical execution were documented. Due to the 4-period replicated crossover design, subjects who prematurely discontinued the study but completed at least one Test and one Reference period provided evaluable pharmacokinetic data and were retained in the per-protocol (PP) population. The precise reasons for subject discontinuation across the two studies were as follows:

Fasting Study: Six subjects were discontinued, exclusively due to the voluntary withdrawal of their informed consent. Ultimately, 28 subjects completed all 4 clinical periods, while 34 subjects provided evaluable data for the PP dataset.

Fed Study: Ten subjects were eliminated: five voluntarily withdrew their informed consent; three were excluded for protocol non-compliance or meeting elimination criteria (two failures to completely consume the standardized high-fat breakfast and one positive urine drug screening before second period dosing); and two were withdrawn directly secondary to adverse events (one case of vomiting and one case of acid peptic disease). Consequently, while 56 participants physically completed all 4 periods, two additional subjects who discontinued during period 4 contributed evaluable pharmacokinetic data and were retained in the per-protocol analysis. Consequently, the final PP population consisted of 58 subjects.

All subjects were discontinued because of adverse events and received close medical monitoring by the clinical staff until complete symptom resolution was achieved. In strict accordance with the study protocol and applicable regulatory guidelines, these withdrawn participants were excluded from the subsequent pharmacokinetic and bioequivalence statistical analyses.

### 2.6. Exploratory IVIVC Analysis

The predictability of the deconvolution approaches evaluated in the exploratory IVIVC analysis is summarized in Table 6.

Marked differences were observed among the evaluated deconvolution approaches. The Wagner–Nelson method underestimated both C_max_ and AUC_0-t_ for the test and reference products, resulting in mean absolute prediction errors of 35.83% and 72.34%, respectively. Both values substantially exceeded the predefined acceptance criteria.

In contrast, both Loo–Riegelman and numerical deconvolution provided improved predictions. Loo–Riegelman yielded mean absolute prediction errors of 11.75% for C_max_ and 9.42% for AUC_0-t_, whereas numerical deconvolution produced the lowest prediction errors, with corresponding values of 6.79% and 4.66%, respectively. Among the evaluated approaches, numerical deconvolution provided the closest agreement between predicted and observed PK values. Only numerical deconvolution satisfied all predefined predictability criteria, with individual prediction errors below 15% and mean absolute PE below 10% for C_max_ and AUC_0-t_.

**Figure 4:**
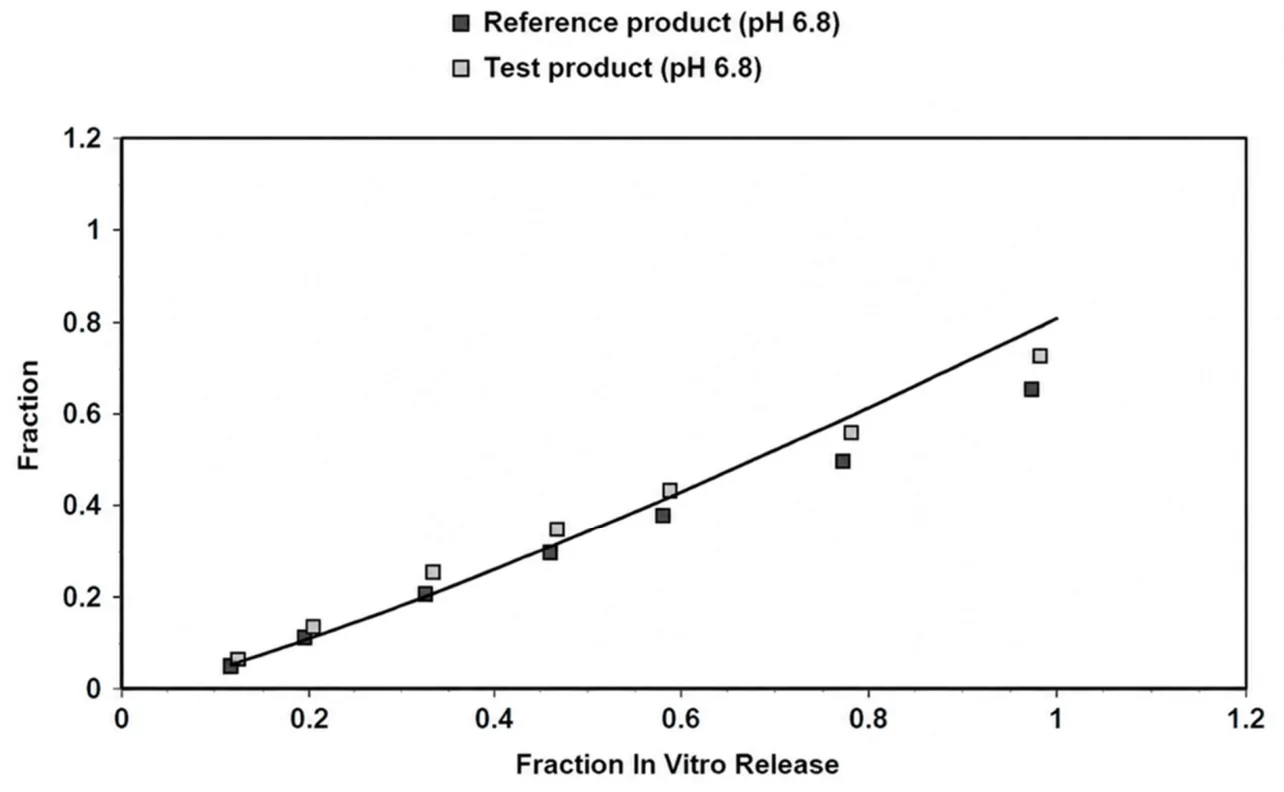
Exploratory in vitro–in vivo relationship for the test and reference extended-release tamsulosin formulations using dissolution data obtained at pH 6.8 and numerical deconvolution.

Overall, the exploratory IVIVC analysis identified numerical deconvolution as the approach that best described the relationship between in vitro dissolution and in vivo absorption for the extended-release tamsulosin formulations evaluated in this study. To visually illustrate this relationship, a correlation plot based on the numerical deconvolution model is presented in Figure 4. This graphical representation plots the cumulative in vivo fraction absorbed against the in vitro fraction dissolved, demonstrating a robust linear correlation that further supports the predictive capability of the selected model.

## 3. Discussion

The primary objective of this study was to establish bioequivalence and evaluate the in vitro–in vivo correlation of a generic extended-release tamsulosin formulation. Given that tamsulosin remains a cornerstone alpha-1 adrenergic antagonist for the management of LUTS associated with BPH [20,21,22,23], demonstrating precise pharmacokinetic control is critical. Our clinical results confirm that the evaluated matrix system successfully maintains the required prolonged drug release, effectively blunting the C_max_. This controlled pharmacokinetic profile is essential to prevent the systemic vasodilatory adverse effects—such as orthostatic hypotension—commonly associated with immediate-release alpha-blockers, thereby ensuring both safety and clinical convenience of a once-daily regimen [24].

Historically, conventional modified-release (MR) formulations have faced limitations, including susceptibility to variations in gastrointestinal transit and a pronounced “food effect” that alters absorption rates [25]. The evaluation under both dietary states is particularly relevant for extended-release systems. Unlike immediate-release tablets, ER matrices are generally more susceptible to food-effect variations, including alterations in gastrointestinal transit time, luminal pH, and mechanical stress [26]. A major clinical and regulatory concern is the potential for “dose dumping”—the rapid, unintended release of the entire dose induced by the presence of a high-fat meal, which can lead to severe hypotensive episodes [24,27].

More advanced delivery technologies, such as the oral controlled absorption system (OCAS), were developed to overcome these challenges by employing an independent, fluid-gel matrix that ensures a consistent, order-zero release profile throughout the entire intestinal tract, including the colon [28].

This technology was specifically designed to minimize the impact of gastrointestinal transit and food intake on drug absorption by providing pH-independent drug release and sustained delivery throughout the gastrointestinal tract and has been recognized as a benchmark for extended-release tamsulosin formulations [28].

In this context, the pharmacokinetic performance of the generic tamsulosin 0.4 mg ER formulation evaluated in the present work is significant. Although our formulation uses a conventional ER matrix rather than OCAS technology, bioequivalence was demonstrated under both fasting and fed conditions, with a favorable safety and tolerability profile. These findings indicate that comparable systemic exposure and clinical performance can be achieved without necessarily employing the same drug-delivery technology, provided that controlled drug release and consistent absorption characteristics are maintained [29]. The close correspondence between these historical values and the metrics obtained in the present study confirms the ability of the generic formulation to maintain stable therapeutic levels throughout the 24 h dosing interval, providing no evidence of abrupt drug release consistent with dose dumping) [30].

The mechanistic basis for this absence of food-induced dose dumping lies in the formulation’s structural architecture and its interaction with postprandial gastrointestinal physiology. A standardized high-fat, high-calorie meal induces massive physiological changes: it significantly delays gastric emptying, prolongs gastric retention time, alters luminal pH, stimulates the secretion of bile salts, and generates intense mechanical and hydrodynamic shear forces due to gastric churning [14,26,31]. Upon ingestion, the hydrophilic polymer matrix of the generic formulation rapidly hydrates to form a highly viscous, protective gel layer around the core [27]. This robust gel barrier exhibits high mechanical strength, allowing it to withstand the intense hydrodynamic conditions and crushing forces of the fed stomach without premature disintegration [12,27]. Furthermore, because the primary release-controlling polymers are predominantly non-ionic, the drug release kinetics remain largely pH-independent, preventing abrupt drug dumping despite the dynamic pH shifts that occur from the fed stomach to the duodenum [26,27]. Finally, while the postprandial release of bile salts acts as endogenous surfactants that can erode lipophilic matrices or alter the solubility of certain drugs, the highly cross-linked hydrophilic gel layer of this matrix is inherently resistant to surfactant-mediated erosion [13,26]. Consequently, the formulation safely transitions through the gastrointestinal tract, maintaining its extended-release integrity regardless of the profound physiological stress induced by food.

When comparing our pharmacokinetic data against published OCAS profiles [24,28], some differences in peak exposure become apparent. The C_max_ values observed in the present study were generally higher than those reported in several OCAS studies, where values typically range from 5.0 to 8.0 ng/mL [24,28]. Despite this difference in peak exposure, the time to reach peak concentration (t_max_) remains comparable to the OCAS benchmark, generally falling within the 5.0–7.0 h range [24,28]. Although relatively flat concentration-time profiles characterize OCAS formulations, the evaluated formulation also maintained controlled absorption and a favorable safety profile despite exhibiting somewhat higher peak concentrations. The absence of an abrupt increase in peak exposure, together with the prolonged t_max_ observed under both dietary conditions, supports the matrix’s structural robustness and its ability to maintain controlled drug release despite the physiological changes associated with food intake [7,32].

The exploratory food-effect assessment provided further insight into these observations. Food intake increased systemic exposure in both the Test and Reference formulations, as reflected by higher C_max_ and AUC values under fed conditions. Nevertheless, these findings should be interpreted within the context of the study design and the characteristics of the evaluated formulations. The fasting and fed investigations were conducted as two independent bioequivalence studies rather than as a dedicated crossover food-effect study; therefore, the Fed/Fasting comparisons are descriptive and should not be interpreted as a formal assessment of food effect. Furthermore, the evaluated formulations are based on different extended-release technologies, with the Test product employing a standard extended-release matrix and the Reference product incorporating the OCAS, which was specifically developed to minimize food-related pharmacokinetic variability [28]. Importantly, despite the observed increase in systemic exposure, food intake was associated with a prolonged rather than shortened t_max_ in both formulations, indicating preservation of the controlled-release absorption profile. Taken together, these findings support the conclusion that the formulation’s controlled-release characteristics were maintained under fed conditions and are inconsistent with food-induced dose dumping. Our regional data comparisons corroborate these pharmacokinetic findings. For instance, in a crossover study evaluating fixed-dose combinations of tamsulosin and dutasteride in healthy Brazilian subjects [33], the reference formulations exhibited C_max_ values of ~12.4 ng/mL under fasting conditions and ~11.3 ng/mL under fed conditions. The close correspondence between these literature values and our findings (with a t_max_ between 5.5 and 6.0 h and a t_1/2_ of 11–12 h) suggests low interethnic variability in tamsulosin disposition among Latin American populations. These observations further support the reliability of our formulation’s release performance.

In addition to primary exposure metrics, secondary pharmacokinetic parameters were evaluated to fully characterize the absorption and disposition kinetics of the extended-release formulations. The prolonged mean residence time (MRT), which averaged between 20.4 and 22.0 h across both prandial states, confirms the sustained absorption profile characteristic of extended-release delivery systems, differentiating it from the rapid absorption typically seen with immediate-release counterparts [7,10].

The apparent clearance (CL/F) in our study ranged from 2.03 to 2.93 L/h, which aligns perfectly with the established pharmacokinetic profile of tamsulosin [9,10]; for instance, Franco-Salinas et al. [10] reported a comparable apparent clearance of approximately 2.4 L/h in healthy subjects. Furthermore, the apparent volume of distribution (Vd/F) ranged from 30.0 to 49.9 L, consistent with the extensive tissue distribution and high bioavailability previously documented for this drug [9]. The apparent terminal half-life (t_1/2_) remained remarkably consistent at 11.7 to 13.5 h across both dietary conditions and treatments. This t_1/2_ is consistent with the 10–15 h range classically reported for modified-release tamsulosin [10]. Notably, given that the intrinsic intravenous half-life of tamsulosin is approximately 5.5 h [9], this prolonged apparent t_1/2_ observed in our study is indicative of flip-flop kinetics. Interestingly, intrinsic flip-flop kinetics are predominantly associated with poorly permeable Biopharmaceutics Drug Disposition Classification System (BDDCS) Class 3 and 4 drugs [34]. Tamsulosin, however, is a highly metabolized drug [34], falling outside these classes. This further confirms that the flip-flop phenomenon observed here is not an inherent property of the active pharmaceutical ingredient but is strictly formulation-driven. It reflects the continuous and sustained absorption of the drug from the matrix throughout the lower gastrointestinal tract, which is the hallmark of its successful extended-release design [10]. Furthermore, despite the inherent complexities of gastrointestinal transit and the potential for food-drug interactions with matrix formulations, the intra-subject variability (CVintra) for systemic exposure remained moderate, ranging from 17.4% to 26.2%. This is highly consistent with the reproducible absorption kinetics reported for advanced delivery systems such as OCAS [28].

Across these two independent clinical studies conducted in healthy Mexican male volunteers—under strictly controlled fasting and fed conditions—the primary pharmacokinetic parameters (C_max_, AUC_0-t_, and AUC_0-∞_) met the regulatory acceptance criteria of 80.00% to 125.00%. The bioequivalence demonstrated here supports the therapeutic equivalence and safe interchangeability of the evaluated generic formulation.

The dissolution profiles of the test and reference extended-release tamsulosin formulations were highly comparable, as evidenced by f_1_ = 2.04 and f_2_ = 92.47. These results indicate a very similar in vitro release behavior between formulations and are consistent with the bioequivalence demonstrated under both fasting and fed conditions. The gradual and controlled release observed throughout the dissolution study is also consistent with the extended-release design of both products. It supports the absence of relevant differences in drug-release performance.

Although the dissolution method used in the exploratory IVIVC analysis corresponds to the quality-control procedure used for routine batch release testing, the exploratory analysis demonstrated that it could provide relevant information on the relationship between in vitro drug release and in vivo absorption. This finding is noteworthy, given that pharmacopeial dissolution methods are primarily intended for quality control and may not always provide a reliable basis for establishing quantitative in vitro–in vivo relationships [35]. Nevertheless, when combined with appropriate deconvolution techniques, the dissolution obtained under the selected experimental conditions provided a useful description of the relationship between in vitro drug release and in vivo absorption.

A limitation of the present in vitro analysis is the reliance on a single pharmacopeial quality-control dissolution condition (pH 6.8 phosphate buffer). While this specific condition was sufficient to establish in vitro profile similarity (f_2_ = 92.47) and successfully support the exploratory IVIVC using numerical deconvolution, the dissolution data were generated under routine pharmacopeial quality-control conditions, which were appropriate for comparing the in vitro performance of the evaluated formulations and supporting the exploratory IVIVC analysis, but were not intended to establish a regulatory dissolution methodology. Consequently, it does not fully replicate the complex physicochemical and hydrodynamic environment of the human gastrointestinal tract [27].

The Wagner–Nelson method produced large prediction errors for both C_max_ and AUC_0–t_ and failed to meet the predefined criteria for predictability. Loo–Riegelman improved predictive performance, particularly for AUC_0–t,_ but did not meet all predefined acceptance criteria because the prediction error for the test formulation’s C_max_ was outside the predefined acceptance range. In contrast, numerical deconvolution was the only approach that met all acceptance criteria, with individual prediction errors below 15% and mean absolute prediction errors below 10% for C_max_ and AUC_0-t_. Moreover, this approach provided the closest agreement between predicted and observed pharmacokinetic values.

These findings suggest that, for extended-release tamsulosin formulations, numerical deconvolution may better characterize the complex absorption kinetics associated with controlled drug release than traditional compartment-based approaches. Importantly, this predictive performance was achieved using dissolution profiles generated under routine quality-control conditions, highlighting the potential utility of numerical deconvolution as a practical tool for early biopharmaceutical assessment and formulation development.

Previous work by Park et al. reported successful development of an IVIVC for modified-release tamsulosin formulations using the Wagner–Nelson method [36]. Interestingly, the dissolution conditions in that study were essentially identical to those in the present investigation, namely, USP Apparatus II at 100 rpm in phosphate buffer (pH 6.8). In both studies, dissolution data generated under these conditions were used to describe in vivo drug absorption, suggesting that this dissolution methodology may have bio-predictive value for extended-release tamsulosin formulations. However, important methodological differences existed in the in vivo component and IVIVC evaluation strategy. Park et al. developed their IVIVC using pharmacokinetic data from Beagle dogs. The present study used human pharmacokinetic data obtained from a fasting bioequivalence study and assessed predictability using predefined PE criteria for C_max_ and AUC_0–t_. These differences in study design and validation approach may account for the different conclusions reached regarding the performance of the deconvolution methods.

The results obtained with numerical deconvolution suggest that this approach may be particularly useful for exploratory IVIVC assessments involving extended-release tamsulosin formulations. However, the present analysis should not be interpreted as the development of a regulatory Level A IVIVC. The study was based on dissolution and pharmacokinetic data obtained from a limited number of formulations, and external validation was not performed. Consequently, the results should be viewed as exploratory in nature and intended to investigate the potential biopredictive capability of the dissolution method rather than to establish a validated regulatory IVIVC model. Nevertheless, the findings suggest that this approach may serve as a useful starting point for future IVIVC development and for guiding the development of generic extended-release tamsulosin drug products.

While the current numerical deconvolution model demonstrated acceptable internal predictive performance, the present correlation should not be interpreted as a regulatory Level A IVIVC. Nevertheless, the findings support the potential utility of numerical deconvolution as an exploratory tool for investigating the relationship between in vitro dissolution and in vivo absorption in extended-release tamsulosin formulations. Further studies, including external validation, would be required before considering its use to support future formulation development or regulatory applications such as IVIVC-based biowaivers.

Taken together, the findings indicate that dissolution profiles generated under routine quality-control conditions can provide relevant information regarding the in vivo performance of extended-release tamsulosin formulations when appropriate deconvolution methods are applied. Among the evaluated approaches, numerical deconvolution provided the most accurate representation of the relationship between in vitro dissolution and in vivo absorption.

The safety and tolerability findings offer robust clinical support for the pharmacokinetic observations, confirming the favorable performance of the extended-release (ER) formulation. While a numerically higher frequency of adverse events (AEs) was recorded during the fed study (32 events) compared to the fasting study (8 events), this discrepancy is primarily attributable to the physiological impact of the standardized high-fat, high-calorie meal rather than systemic drug toxicity [31,37]; indeed, events in the fed state—such as nausea, dyspepsia, abdominal pain, and vomiting—reflect transient gastrointestinal intolerance to the diet, whereas the most common systemic events observed across both studies, headache and dizziness, align with the established pharmacological profile of α1-blockers [29]. Crucially, the low incidence of hemodynamic AEs and the complete absence of clinically significant orthostatic hypotension indicate that the ER mechanism successfully prevented toxic systemic exposures. Furthermore, participant discontinuations across both studies were predominantly due to voluntary withdrawal of consent or protocol non-compliance, with only 2 withdrawals directly related to adverse gastrointestinal events.

The safety and tolerability findings offer robust clinical support for the pharmacokinetic observations, particularly regarding the exposure-response relationship typical of $\alpha_{1}$-blockers. The systemic vasodilatory adverse events of tamsulosin, such as orthostatic hypotension, dizziness, and reflex tachycardia, are primarily concentration-driven and closely linked to acute spikes in peak plasma exposure (C_max_) [7,10]. In both clinical trials, the complete absence of symptomatic orthostatic hypotension or clinically significant hemodynamic shifts demonstrates that the extended-release mechanism effectively blunted the C_max_ to safely tolerated levels. This is strongly supported by the continuous vital signs monitoring, which showed a maximum mean systolic blood pressure drop of less than 5 mmHg (from 113.3 to 109.0 mmHg) at the time points coinciding with peak plasma exposure (t_max_), remaining completely stable through the end of the 24 h dosing period. Furthermore, mean heart rates remained entirely within normal physiological limits—transitioning safely from an early morning baseline of 61.1 bpm to expected daytime activity peaks averaging 82.0 bpm, and returning smoothly to baseline levels (~67.0 to 74.5 bpm) at the completion of the 24 h dosing cycle—with no clinical evidence of drug-induced reflex tachycardia. This highly stable post-dose hemodynamic profile indicates that the generic formulation’s release kinetics successfully maintain plasma concentrations within a therapeutic window that minimizes cardiovascular toxicity, an outcome that aligns closely with the established hemodynamic safety of reference modified-release tamsulosin systems [15,24].

These findings provide strong evidence that our formulation strikes a favorable balance between robust systemic exposure and patient safety. By demonstrating bioequivalence to the innovator product while maintaining a safety profile that rivals that of OCASsystems, our formulation establishes itself as a reliable and therapeutically equivalent alternative. The safety data—specifically, the absence of serious adverse events and the mild nature of reported events—strongly support the robustness of the formulation’s ER mechanism and its suitability for managing LUTS/BPH in the target population.

Finally, from a metabolic perspective, tamsulosin undergoes extensive hepatic metabolism via CYP2D6 and CYP3A4 [38]. While genetic polymorphisms in these enzymes can influence systemic exposure, the crossover design of our studies effectively controlled for inter-subject metabolic variance. The absence of significant sequence or period effects in the ANOVA suggests that bioequivalence is a function of formulation design rather than individual metabolic phenotypes.

Like most regulatory bioequivalence assessments, these studies were conducted under single-dose conditions in a healthy, relatively young demographic. While this provides a rigorous, controlled, and sensitive model to isolate the release performance of the formulations, it may not fully capture the clinical reality of the target demographic. Patients with benign prostatic hyperplasia (BPH) are predominantly elderly individuals who frequently present with age-related physiological changes, such as reduced gastrointestinal motility and decreased hepatic blood flow [39]. These factors, combined with highly prevalent comorbidities and polypharmacy—such as combination therapies with 5-alpha-reductase inhibitors or antimuscarinics—can significantly alter tamsulosin’s hepatic metabolism and renal excretion [10,39]. Drug–drug interactions, particularly those involving CYP3A4 and CYP2D6 inhibitors common in geriatric polypharmacy, may reduce systemic clearance and increase overall exposure [10]. However, the robust performance of the extended-release matrix evaluated in this study becomes especially clinically relevant for this vulnerable population. Because standard tamsulosin therapy carries known risks of postural hypotension and dizziness [39], the formulation’s blunted C_max_ inherently protects against acute hypotensive spikes that might otherwise be exacerbated by age-related pharmacokinetic alterations [24].

Ultimately, the present research provides robust pharmacokinetic, statistical, and clinical evidence demonstrating that the evaluated tamsulosin 0.4 mg ER formulation is bioequivalent, safe, and interchangeable. By achieving pharmacokinetic stability and a safety profile comparable to those of advanced delivery systems, this formulation offers a reliable and efficacious therapeutic alternative for managing LUTS/BPH in Latin American populations.

Looking forward, the successful exploratory IVIVC demonstrated in this study lays the groundwork for more advanced computational approaches in formulation development. Future research directions should further investigate the relationship between in vitro dissolution and in vivo performance by incorporating physiologically based pharmacokinetic (PBPK) and physiologically based biopharmaceutics modeling (PBBM). Such model-informed approaches may also contribute to future advances in virtual bioequivalence trials for modified-release formulations. However, additional external validation will be required before these approaches can be considered for broader scientific or regulatory applications.

## 4. Materials and Methods

### 4.1. Drug Products

The investigational test product consisted of generic extended-release (ER) tamsulosin hydrochloride 0.4 mg capsules, developed and manufactured by Monteverde S.A. The innovator reference product used for comparison was Omnic Ocas^®^ (tamsulosin hydrochloride 0.4 mg) ER capsules, manufactured by Astellas Pharma Europe B.V. (Meppel, The Netherlands) and commercially obtained from the authorized Brazilian market.

The reference product was identified as the originator formulation, which is the recognized reference drug approved based on a full registration dossier (including complete preclinical and clinical data). This classification was confirmed using the official Reference Drug List published by ANVISA [40]. Certificates of Analysis (CoAs) for both the test and reference products were obtained directly from their respective manufacturers prior to study initiation, confirming compliance with pharmacopeial specifications. Throughout the duration of the study, all drug products were stored in their original, intact packaging under controlled temperature and humidity conditions to preserve their physicochemical integrity.

Both the test and reference formulations were administered as prolonged-release capsules. The generic test product (tamsulosin hydrochloride 0.4 mg ER capsules) was developed as a system designed to provide continuous, extended drug release over a 24 h period, minimizing peak-related systemic exposure. The formulation incorporates a primary release-controlling polymer.

The manufacturing process involves a strategy specifically selected to ensure uniform drug distribution and optimal matrix integrity. During pharmaceutical development, the formulation optimization strategy focused on modulating the polymer-to-excipient ratio to mimic the target absorption profile and ensure structural robustness against gastrointestinal hydrodynamic stress. The Critical Quality Attributes (CQAs) rigorously monitored during batch release included drug assay and uniformity of dosage units, guaranteeing consistent controlled-release performance prior to clinical evaluation.

Before clinical administration, the batch numbers, manufacturing dates, and expiration dates of both the test and reference products were rigorously verified. A comprehensive review of the Certificates of Analysis (CoA) confirmed that the test batch fully complied with the pre-established quality specifications, including assay, uniformity of dosage units, and in vitro dissolution profiles, as mandated by the Mexican regulatory guideline NOM-177-SSA1-2013 for investigational products intended for bioequivalence studies [41].

To ensure the physical and chemical stability of the formulations, all study medications were stored in a secure, access-controlled pharmacy under strictly monitored environmental conditions (temperature between 15 °C and 30 °C, protected from moisture and direct light), in full accordance with the manufacturer’s labeled instructions and Good Clinical Practice (GCP) guidelines, until the time of dispensing.

### 4.2. In Vitro Dissolution Procedure

The dissolution profiles used in this analysis were obtained from the same test and reference batches evaluated in the fasting bioequivalence study reported herein. Dissolution testing was performed using USP Apparatus II (Classic 6 dissolution tester, Hanson Research, Chatsworth, CA, USA). Sinkers were used to prevent the dosage forms from floating during testing.

The dissolution testing was conducted in 900 mL of 50 mM potassium phosphate buffer (pH 6.8), prepared using 6.8 g/L of KH_2_PO_4_ and 0.896 g/L NaOH, with the pH adjusted to 6.8 ± 0.05 using concentrated phosphoric acid at 37.0 ± 0.5 °C with a paddle rotation speed of 100 rpm. Samples were collected at 1, 2, 4, 6, 8, 12, and 20 h without medium replacement. Before analysis, dissolution samples were filtered through 0.45 µm nylon membrane filters. The amount of dissolved tamsulosin was quantified at 225 nm by HPLC using a Prominence LC-20AD SIL-20AC system coupled to an SPD-10A UV detector (Shimadzu, Kyoto, Japan) and a Luna C_18_ column (150 mm × 4.6 mm, 5 µm; Phenomenex^®^, Torrance, CA, USA).

### 4.3. Food-Effect Assessment

To further characterize the influence of food on the pharmacokinetic performance of the extended-release tamsulosin formulations, a descriptive food-effect assessment was conducted using pharmacokinetic data from independent fasting and fed bioequivalence studies. Arithmetic mean values of C_max_, AUC_0-t_, and AUC_0-∞_ obtained under both dietary conditions were compared and fed-to-fasting (Fed/Fasting) ratios were calculated for each parameter. This exploratory analysis was intended to characterize the effect of food on systemic drug exposure and to descriptively assess whether the observed pharmacokinetic behavior was consistent with the expected performance of an extended-release formulation, thereby providing supportive evidence for the subsequent discussion of the potential risk of food-induced dose dumping. Because the fasting and fed studies were conducted as independent bioequivalence trials rather than as a dedicated crossover food-effect study, no formal statistical comparison or predefined acceptance criteria were applied to the Fed/Fasting comparisons.

### 4.4. Studies Design

The clinical protocols and informed consent documents were thoroughly reviewed and approved by an independent Research Ethics Committee and the Federal Commission for the Protection Against Sanitary Risks (COFEPRIS) in Mexico. Both clinical trials were conducted in strict adherence to the ethical principles outlined in the Declaration of Helsinki (including the 75th WMA General Assembly amendments, October 2024) [42] and the International Council for Harmonization (ICH) guidelines for Good Clinical Practice (GCP) [43].

To comprehensively evaluate the pharmacokinetic profile of the tamsulosin formulations, two separate single-center, single-dose, open-label, randomized, four-period, two-sequence crossover studies were conducted: one under fasting conditions and another under fed conditions. The clinical execution of both trials was carried out by Axis Clinicals Latina, S.A. de C.V. (Mexico City, Mexico).

For the fasting study (n = 34 randomized subjects), participants underwent a supervised overnight fast of at least 10 h before drug administration and continued fasting for an additional 4 h post-dose. For the fed study (n = 66 randomized subjects), after an identical 10 h overnight fast, volunteers were served a standardized high-fat, high-calorie breakfast (approximately 800–1000 kcal). Participants were required to consume this meal in full within 30 min before dosing to adequately evaluate the food effect.

During each clinical period, subjects were confined to the clinical facility from the evening before dosing (Day 0) until the initial 24 h pharmacokinetic blood sampling was completed. Subsequent blood samples were collected during ambulatory return visits up to 72 h post-administration. A washout period of 7 days separated the two treatment periods to ensure complete drug elimination and prevent carry-over effects. Throughout the confinement and ambulatory phases, medical personnel continuously monitored participants’ vital signs and rigorously documented any adverse events, including their severity, duration, and causality.

### 4.5. Study Population

Participation in these trials was strictly voluntary. Written informed consent was obtained from all participants before their enrollment and the initiation of any protocol-specific screening procedures. Given the clinical indication of the investigational product, the study population comprised exclusively healthy Mexican male volunteers aged 18 to 55 years, with a body mass index (BMI) maintained between 18.0 and 27.0 kg/m^2^, in compliance with the demographic requirements established by the Mexican regulatory standard NOM-177-SSA1-2013 [41].

To confirm participants’ health status, a comprehensive clinical screening was conducted within 14 days before the first dose. This assessment included a detailed medical history, a full physical examination, a resting 12-lead electrocardiogram (ECG), a complete panel of laboratory tests (hematology, blood chemistry, and urinalysis), and serology and urine drug screens.

Upon admission to the clinical unit for Period 1 (Day 0), eligibility was reassessed, and confirmatory breath alcohol and urine drug tests were administered. Key exclusion criteria for both studies included: any documented history of cardiovascular, hepatic, renal, or gastrointestinal pathologies that could alter drug absorption or disposition; a history of drug or alcohol abuse; participation in another clinical trial within the previous 90 days; and the use of any prescription or over-the-counter medications within 14 days before the investigational product administration.

### 4.6. Sample Size

In accordance with the regulatory framework outlined in the NOM-177-SSA1-2013 guidelines [41], the estimation of statistical power and sample size must be based on the pharmacokinetic parameter with the highest intra-subject coefficient of variation (CV_intra_). Reference data derived from an unpublished internal pilot trial conducted by Axis Clinicals Latina indicated peak CV_intra_ values of 46.00% for AUC_0-t_ and 40.00% for C_max_ under fed and fasting conditions, respectively.

Sample size computations and subsequent statistical analyses were performed using R software, version 4.1.2 [44]. The calculations were based on the two-one-sided test (TOST) procedure, specifically designed for a 2 × 2 × 4 (2 sequence, 2 treatments, and 4 periods) crossover design [45]. The calculation was based on stringent predefined parameters: an alpha level of 5% (α = 0.05), a target power of at least 80%, and an anticipated test-to-reference geometric mean ratio of 0.95.

Applying these statistical boundaries, demonstrating bioequivalence within the standard 80.00% to 125.00% regulatory acceptance interval required a minimum of 28 evaluable subjects for the fed study and 58 for the fasting study. To proactively mitigate the statistical impact of anticipated dropouts, protocol deviations, or premature participant withdrawals, the final enrollment targets were conservatively adjusted upwards to 36 and 70 randomized volunteers for the fed and fasting studies, respectively.

### 4.7. Pharmacokinetic Assessment and Analytical Method

To accurately characterize the pharmacokinetic profiles of the formulations, sequential venous blood draws (approximately 6 mL each) were collected in Vacutainer tubes containing sodium heparin as the anticoagulant. Sampling was performed before drug administration (0 h, baseline) and at the following twenty post-dose intervals: 0.50, 1.00, 2.00, 3.00, 3.50, 4.00, 4.50, 5.00, 5.50, 6.00, 6.50, 7.00, 8.00, 10.00, 12.00, 16.00, 24.00, 36.00, 48.00, and 72.00 h.

Immediately following extraction, the blood samples underwent centrifugation (3500 rpm for 10 min at 4 °C) to isolate the plasma fraction. The resulting plasma aliquots were decanted into pre-coded cryotubes and stored at −70 ± 15 °C to ensure analyte stability until laboratory analysis.

The in vivo plasma concentrations of tamsulosin were determined utilizing a highly sensitive and selective liquid chromatography-tandem mass spectrometry (LC-MS/MS) system. The bioanalytical methodology was internally developed and extensively validated by the analytical unit at Axis Clinicals Latina, S.A. de C.V. (Mexico City, Mexico). This comprehensive validation process was conducted in accordance with Good Laboratory Practice (GLP) frameworks and prevailing Mexican regulatory norms, while also considering internationally recognized bioanalytical method validation guidelines, including those established by the U.S. Food and Drug Administration (FDA) [46].

Sample preparation was performed using a liquid–liquid extraction (LLE) technique with tamsulosin-d4 hydrochloride as the internal standard (IS).

Briefly, 300 µL of human plasma was transferred into glass tubes containing 50 µL of the IS solution and 400 µL of pretreatment solution. After 10 s of vortex mixing, 3 mL of extraction solvent was added, and the samples were agitated for 10 min at 2000 rpm. Subsequently, the samples were centrifuged for 5 min at 3500 rpm and 5 °C to achieve phase separation. The organic phase was then collected and evaporated under a stream of nitrogen at 50 °C for 20 min. The resulting residue was reconstituted in 500 µL of mobile phase and transferred into autosampler vials before chromatographic analysis.

Chromatographic separation was performed using a Prominence LC-20AD/SIL-20AC high-performance liquid chromatography system (Shimadzu, Kyoto, Japan) equipped with a Kinetex^®^ C_8_ column (100 mm × 4.6 mm, 2.6 µm, 100 Å; Phenomenex, Torrance, CA, USA) protected by a Phenomenex C_18_ guard column (4 mm × 3 mm). The mobile phase consisted of an organic phase and 1 mM ammonium formate buffer (pH 3.0 ± 0.3) in an 85:15 (*v*/*v*) ratio, delivered at a flow rate of 0.85 mL/min. The autosampler and column temperatures were maintained at 10 °C and 40 °C, respectively. A 20 µL aliquot was injected into the chromatographic system.

Detection was carried out using an API 4000 triple quadrupole mass spectrometer (AB Sciex, Singapore) coupled to a TurboIonSpray interface operating in positive electrospray ionization mode (ESI+). Quantification was achieved by multiple reaction monitoring (MRM). The monitored precursor-to-product ion transitions were *m*/*z* 409.2→228.2 for tamsulosin and *m*/*z* 413.2→228.1 for tamsulosin-d4.

Calibration standards covered a concentration range of 0.100–40.531 ng/mL. Quality control (QC) samples were prepared at concentrations of 0.298, 13.250, and 30.814 ng/mL, representing the low-, medium-, and high-concentration regions of the calibration range. Quantification was carried out using Analyst^®^ software (version 1.6.2) by linear regression of analyte-to-internal-standard peak area ratios against nominal concentrations, with a 1/x^2^ weighting factor applied to the calibration model.

The validated method demonstrated satisfactory selectivity, linearity, precision, accuracy, sensitivity, recovery, matrix effect, and stability throughout the validated concentration range. The method met the predefined acceptance criteria for selectivity, linearity, lower limit of quantification (LLOQ), precision, accuracy, recovery, matrix effect, carry-over, and stability in accordance with current regulatory recommendations for bioanalytical method validation. A detailed summary of the bioanalytical method validation, including calibration performance, predefined acceptance criteria, quantitative validation results, precision, accuracy, selectivity, matrix effect, recovery, sensitivity, carry-over, LLOQ and stability assessments, is provided in the Supplementary Materials.

### 4.8. Tolerability and Safety Assessments

The clinical safety and tolerability profiles of the investigational products were continuously scrutinized throughout the confinement periods. Comprehensive physical examinations were performed by qualified medical staff at screening, upon admission, and immediately before the subject’s final clinical discharge.

To safeguard participant well-being and closely monitor the expected hemodynamic effects associated with α1-blockers, vital signs—specifically blood pressure, heart rate, and body temperature—were systematically measured at baseline (hour 0) and at prespecified post-dose intervals: 7.00, 12.00, 24.00, 36.00, 48.00, and 72.00 h.

The incidence, severity, and potential relationships between adverse events (AEs) and the administered formulations were rigorously documented. AEs were identified through both spontaneous reporting by subjects and active clinical surveillance by the investigating physicians. Each recorded event was formally categorized by severity (mild, moderate, or severe), duration, need for medical intervention, and final clinical outcome, thereby establishing a definitive and highly robust tolerability profile for the evaluated tamsulosin treatments.

### 4.9. Exploratory In Vitro–In Vivo Correlation Analysis

To further explore the relationship between in vitro drug release and in vivo absorption, an exploratory in vitro–in vivo correlation (IVIVC) analysis was conducted using dissolution and pharmacokinetic data obtained in the present study under fasting conditions. The objective of this analysis was not to establish a regulatory IVIVC model, but rather to investigate whether dissolution profiles obtained under routine quality-control conditions could provide meaningful information regarding the in vivo absorption behavior of extended-release tamsulosin formulations. In addition, the predictive performance of different deconvolution approaches was evaluated. Mean dissolution profiles of the test and reference formulations were calculated and used as the in vitro input for all IVIVC analyses.

The in vivo input consisted of the mean plasma concentration–time profiles obtained under fasting conditions. Three deconvolution approaches commonly employed for IVIVC development were evaluated: the Wagner–Nelson method, the Loo–Riegelman method, and numerical deconvolution. All modeling and deconvolution analyses were performed using GastroPlus^®^ version 9.9 (Simulations Plus Inc., Lancaster, CA, USA).

An intravenous (IV) disposition model was developed using published pharmacokinetic data from van Hoogdalem et al. in 10 healthy male volunteers following IV infusion of 0.125 mg tamsulosin hydrochloride over 4 h [9]. One-, two-, and three-compartment disposition models were evaluated. The two-compartment model provided the most appropriate description of the IV pharmacokinetic profile, based on visual inspection of the fitted profiles, the coefficient of determination (R^2^ = 0.997), and the Akaike Information Criterion (AIC = −246.07), and was therefore selected as the reference disposition model for subsequent analyses. The final model yielded a total clearance (CL) of 3.977 L/h, a total volume of distribution (V_d_) of 15.212 L, and an elimination half-life (t_1/2_) of 5.41 h. The corresponding micro constants were K_10_ = 0.237 h^−1^, K_12_ = 0.155 h^−1^, and K_21_ = 0.311 h^−1^. These estimates were consistent with published intravenous pharmacokinetic data for tamsulosin [9].

The Wagner–Nelson method was implemented using a one-compartment disposition model, as required by its underlying assumptions [47]. Loo–Riegelman deconvolution was performed using the micro constants obtained from the final two-compartment model [48]. Numerical deconvolution was performed using the IV disposition function generated from the selected two-compartment model as the unit impulse response function.

For each approach, the fraction absorbed in vivo (F_abs_) was estimated from the observed oral plasma concentration-time profile and correlated with the fraction dissolved in vitro (F_diss_). The predictive performance of each method was evaluated through internal validation using prediction errors (PE) for C_max_ and AUC_0-t_. Prediction error was calculated as: PE (%) = [(Predicted − Observed)/Observed] × 100 [49].

The predictability of each deconvolution approach was assessed using prediction errors for C_max_ and AUC_0-t_. Mean absolute prediction errors ≤ 10% and individual prediction errors ≤ 15% were considered indicative of acceptable predictive performance [49].

### 4.10. Statistical Analysis

Baseline clinical and demographic parameters for both studies were outlined using standard descriptive statistics. The safety evaluation was performed on the intention-to-treat (ITT) population, comprising all randomized volunteers who received at least one dose of the investigational formulations (n = 34 for fasting; n = 66 for fed).

Pharmacokinetic (PK) and bioequivalence analyses were restricted to the per-protocol (PP) populations. Because the study utilized a robust 4-period replicated crossover design (2 × 2 × 4), the PP population included all subjects who provided quantifiable plasma concentration-time profiles for at least one Test and one Reference period without major protocol deviations, even if they did not physically complete all four clinical periods. After accounting for clinical exclusions, the final evaluable datasets comprised 32 subjects for the fasting study and 58 subjects for the fed study. These sample sizes provided adequate statistical power for the bioequivalence assessment. Notably, subjects who discontinued during later study periods were retained in the PK analysis because sufficient pharmacokinetic data had been collected per the predefined protocol.

The primary pharmacokinetic endpoints mandated for the bioequivalence determination were the peak plasma concentration (C_max_), the area under the plasma concentration-time curve from administration to the last quantifiable sampling point (AUC_0-t_), and the extrapolated area under the curve to infinity (AUC_0-∞_). Secondary kinetic metrics include the time required to achieve maximum concentration (t_max_), the terminal elimination rate constant (K_el_), and the apparent elimination half-life (t_1/2_).

All PK parameters were calculated via non-compartmental analysis (NCA) utilizing Phoenix^®^ WinNonlin^®^ software, version 8.4 (Certara L.P., Princeton, NJ, USA). The C_max_ and t_max_ values were determined directly from the observed plasma concentration-time profiles. The AUC_0-t_ was estimated using the linear trapezoidal method, whereas the AUC_0-∞_ was estimated by extrapolating AUC_0-t_ from the last measurable concentration (C_tlast_) using K_el_.

To statistically confirm bioequivalence, Schuirmann’s two-one-sided tests (TOST) were applied to the natural-log-transformed primary metrics C_max_ and AUC_0-t_. Furthermore, an Analysis of Variance (ANOVA) was conducted to detect statistically significant effects of the treatment sequence, clinical period, or formulation. In strict alignment with the Mexican regulatory guideline NOM-177-SSA1-2013 [41], 90% confidence intervals (CIs) were generated for the geometric mean ratios (Test/Reference). Bioequivalence was conclusively declared if the resultant 90% CIs for C_max_, AUC_0-t_, and AUC^0-∞^ were entirely contained within the predefined regulatory acceptance limits of 80.00% to 125.00%.

For the in vitro dissolution data, the comparison of the dissolution profiles between the test and reference formulations was performed using the model-independent similarity factor (f_2_), in accordance with international regulatory guidelines [41,46,49]. The f_2_ value was calculated using the mean dissolution values at each specified time point. Dissolution profiles were considered when f2 was greater than or equal to 50.

## 5. Conclusions

Ultimately, the present research provides pharmacokinetic, biopharmaceutical, and clinical evidence demonstrating that the evaluated tamsulosin 0.4 mg ER formulation is bioequivalent, safe, and interchangeable. While the fasting and fed evaluations were conducted in independent cohorts—precluding a direct intra-subject statistical comparison of the food effect—the consistent formulation performance and the pharmacokinetic findings, which were not consistent with food-induced dose dumping, suggest that the controlled-release characteristics of the formulation were maintained under both dietary conditions. By satisfying the stringent bioequivalence and in vitro dissolution criteria established not only by the local regulatory authority (COFEPRIS) but also consistent with internationally recognized standards (FDA and EMA), this generic formulation offers a reliable and globally relevant therapeutic alternative for managing LUTS/BPH.

Looking forward, the successful exploratory IVIVC demonstrated in this study lays the groundwork for more advanced computational approaches in formulation development. Future research directions should transcend traditional in vitro and in vivo methodologies by incorporating physiologically based pharmacokinetic (PBPK) modeling and AI-assisted formulation optimization. The integration of machine learning-guided IVIVC predictions holds the potential to revolutionize regulatory science by enabling digital bioequivalence assessments and virtual bioequivalence trials. The adoption of these in silico technologies will not only accelerate the optimization of matrix-based delivery systems but also facilitate the design of personalized dosing strategies, ensuring optimal safety and efficacy profiles for diverse patient populations.

## Figures and Tables

**Table 1:** Summary of baseline demographic characteristics of the healthy Mexican volunteers enrolled in the fasting and fed bioequivalence studies.

Demographic Characteristic	Fasting Study (n = 34)	Fed Study (n = 66)
Age (years)		
Mean ± SD	36.8 ± 10.5	38.5 ± 9.7
Range (min–max)	21.0–54.0	20.0–55.0
Weight (kg)		
Mean ± SD	68.6 ± 7.5	71.2 ± 9.0
Range (min–max)	55.5–88.2	49.7–89.0
Height (m)		
Mean ± SD	1.71 ± 0.07	1.70 ± 0.07
Range (min–max)	1.55–1.83	1.53–1.83
BMI (kg/m^2^)		
Mean ± SD	23.6 ± 2.5	24.5 ± 2.2
Range (min–max)	18.6–27.0	18.9–27.0
Gender, n (%)		
Male	34 (100%)	66 (100%)
Female	0 (0%)	0 (0%)

SD: standard deviation; n: number of subjects; BMI: body mass index; kg: kilograms; m: meters.

**Table 2:** Pharmacokinetic parameters of tamsulosin 0.4 mg extended-release tablets following a single oral dose in healthy Mexican male volunteers under fasting and fed conditions. Data are presented as mean ± standard deviation (SD).

PK Parameter	Tamsulosin (Test)	Tamsulosin (Reference)
Fed study		
C_max_ (ng/mL)	12.73 ± 5.06	13.44 ± 4.81
t_max_ (h)	6.67 ± 2.58	6.61 ± 2.11
AUC_0-t_ (ng·h/mL)	236.30 ± 148.78	235.42 ± 109.62
AUC_0-∞_ (ng·h/mL)	276.50 ± 405.56	257.71 ± 182.91
t_1/2_ (h)	12.08 ± 11.95	11.78 ± 7.21
MRT (h)	20.52 ± 17.74	20.43 ± 11.22
Vd/F (L)	30.03 ± 16.66	30.23 ± 13.01
CL/F (L/h)	2.03 ± 0.85	2.05 ± 1.25
Fasted study		
C_max_ (ng/mL)	8.34 ± 2.35	7.52 ± 2.43
t_max_ (h)	5.56 ± 2.09	5.82 ± 1.65
AUC_0-t_ (ng·h/mL)	162.63 ± 52.53	152.35 ± 57.33
AUC_0-∞_ (ng·h/mL)	177.83 ± 60.26	159.65 ± 59.94
t_1/2_ (h)	13.52 ± 5.48	12.47 ± 3.95
MRT (h)	22.04 ± 7.31	20.83 ± 5.31
Vd/F (L)	45.47 ± 13.88	49.89 ± 19.72
CL/F (L/h)	2.54 ± 0.94	2.93 ± 1.28

C_max_: maximum observed plasma concentration; t_max_: time to reach maximum plasma concentration; AUC_0-t_: area under the plasma concentration-time curve from time zero to the last quantifiable concentration; AUC_0-∞_: area under the plasma concentration-time curve from time zero extrapolated to infinity; t_1/2_: terminal elimination half-life. MRT: mean residence time. Vd/F: apparent volume of distribution. CL/F: apparent total clearance.

**Table 3:** Geometric mean ratios and 90% confidence intervals (CIs) of the primary pharmacokinetic parameters for tamsulosin.

Study/Analyte	Parameter	GMR (%)	90% CI (LL–UL)	CV_intra_ (%)	TOST p1 (<0.80)	TOST p2 (>1.25)	Power
Fed study							
Tamsulosin	C_max_	94.62	89.49–100.40	22.30	<0.001	<0.001	1.00
	AUC_0-t_	99.36	94.14–104.88	24.60	<0.001	<0.001	1.00
	AUC_0-∞_	98.59	93.52–103.95	23.80	<0.001	<0.001	1.00
Fasted study							
Tamsulosin	C_max_	111.50	103.33–120.32	26.20	<0.001	<0.001	1.00
	AUC_0-t_	108.21	100.43–116.58	19.70	<0.001	<0.001	1.00
	AUC_0-∞_	112.62	105.33–120.76	17.40	<0.001	<0.001	1.00

CV_intra:_ Within-subject coefficient of variation; GMR: Geometric Mean Ratio. LL: Lower Limit; UL: Upper Limit. Schuirmann’s Two One-Sided Tests (TOST). C_max_: maximum observed plasma concentration; AUC_0-t_: area under the plasma concentration-time curve from time zero to the last quantifiable concentration.

**Table 4:** Exploratory descriptive comparison of pharmacokinetic parameters under fasting and fed conditions.

PK Parameter	Fasting Test	Fed Test	Fed/Fasting	Fasting Ref	Fed Ref	Fed/Fasting
C_max_ (ng/mL)	8.34	12.73	1.53	7.52	13.44	1.79
t_max_ (h)	5.56	6.67	---	5.82	6.61	---
AUC_0-t_ (ng·h/mL)	162.63	236.30	1.45	152.35	235.42	1.55
AUC_0-∞_ (ng·h/mL)	177.83	276.50	1.55	159.65	257.71	1.61

C_max_: maximum observed plasma concentration; t_max_: time to reach maximum plasma concentration; AUC_0-t_: area under the plasma concentration-time curve from time zero to the last quantifiable concentration; AUC_0-∞_: area under the plasma concentration-time curve from time zero extrapolated to infinity.

**Table 5:** Summary of Adverse Events (AEs) by study and treatment.

Study Condition	Adverse Event	Test Group AEs	Reference Group AEs	Total AEs
Fasting Study	Dizziness	1	2	3
	Headache	1	1	2
	Abdominal pain	1	0	1
	Nasal congestion	1	0	1
	Odynophagia	1	0	1
Fed Study	Headache	10	8	18
	Dizziness	2	3	5
	Nausea	3	0	3
	Pharyngitis	1	1	2
	Vomiting	0	1	1
	Diarrhea	1	0	1
	Acid peptic disease	0	1	1
	Dyspepsia	0	1	1
Total		22	18	40

**Table 6:** Internal validation of the exploratory IVIVC models.

PK Parameter	Observed	Wagner–Nelson Predicted	PE (%)	Loo–Riegelman Predicted	PE (%)	Numerical Deconvolution Predicted	PE (%)
C_max_ Ref (ng/mL)	7.52	5.17	−31.25	6.98	−7.18	7.32	−2.66
C_max_ Test (ng/mL)	8.34	4.97	−40.41	6.98	−16.31	7.43	−10.91
Mean absolute PE (C_max_)	---	---	35.83	---	11.75	---	6.79
AUC_0-t_ Ref (ng·h/mL)	152.35	44	−71.12	143	−6.14	150	−1.54
AUC_0-t_ Test (ng·h/mL)	162.63	43	−73.56	142	−12.69	150	−7.77
Mean absolute PE (AUC_0-t_)	---	---	72.34	---	9.42	---	4.66

C_max_: maximum observed plasma concentration; AUC_0-t_: area under the plasma concentration-time curve from time zero to the last quantifiable concentration. PE: prediction error.

## Data Availability

The data presented in this study are available upon request from the corresponding author due to confidentiality reasons.

## Supplementary Materials

The following supporting information can be downloaded at https://www.mdpi.com/article/10.3390/pharmaceutics18081008/s1. **Table S1**. Measured concentrations and variability across three independent calibration runs for the evaluation of method linearity of tamsulosin in human plasma. **Table S2**. Calibration curve parameters for tamsulosin. **Table S3**. Summary of bioanalytical method validation results for tamsulosin. **Figure S1**. Representative calibration curve obtained using analyte-to-internal standard peak area ratios, showing the linear relationship between concentration and response for tamsulosin in human plasma.

## Abbreviations

The following abbreviations are used in this manuscript:

AEs	Adverse events
ANOVA	Analysis of variance
AUC	Area under the curve
BMI	Body mass index
BPH	Benign prostatic hyperplasia
CBC	Complete blood count
CI	Confidence interval
C_last_	Last measurable plasma concentration
C_max_	Peak plasma concentration
CoA	Certificate of Analysis
COFEPRIS	Federal Commission for the Protection against Sanitary Risk
CRO	Contract Research Organization
CV_intra_	Intra-subject coefficient of variation
CYP	Cytochrome P450
ECG	Electrocardiogram
ER	Extended-release
ESI+	Positive electrospray ionization
FDA	Food and Drug Administration
GCP	Good Clinical Practice
ICH	International Council for Harmonization
IS	Internal standard
ITT	Intention-to-treat
K_el_	Terminal elimination rate constant
LC-MS/MS	Liquid chromatography coupled with tandem mass spectrometry
LL	Lower limit
LUTS	Lower urinary tract symptoms
MRM	Multiple reaction monitoring
NCA	Non-compartmental analysis
OCAS	Oral Controlled Absorption System
OTC	Over-the-counter
PK	Pharmacokinetics
PP	Per-protocol
SAEs	Serious adverse events
SD	Standard deviation
t_1/2_	Elimination half-life
t_max_	Time to reach peak plasma concentration
TOST	Two one-sided tests
UL	Upper limit
