# Supplementary material for: Bioequivalence, Food-Effect Assessment and Exploratory IVIVC of Two Extended-Release Tamsulosin 0.4 mg Formulations in Healthy Mexican Subjects
Source: Pharmaceutics. 2026 Aug 14;18(8):1008. doi: 10.3390/pharmaceutics18081008 (PMC13517056; doi:10.3390/pharmaceutics18081008)
Supplement: Supplementary file 1 [file pharmaceutics-18-01008-s001.zip › pharmaceutics-4485434-supplementary.pdf]

S1. Supplementary material: Bioanalytical Method Validation

Bioanalytical Method Validation for the Determination of Tamsulosin in Human Sodium-Heparin Plasma by LC-MS/MS

S1.1 Objective and regulatory framework

The aim of this study was the complete validation of an analytical method for the determination of tamsulosin in human sodium-heparin plasma.

The bioanalytical method validation was conducted in accordance with the applicable regulatory requirements for bioequivalence studies in Mexico (NOM-177-SSA1-2013). The validation approach is consistent with internationally accepted principles outlined in regulatory guidelines, including those of the European Medicines Agency (EMA) and the U.S. Food and Drug Administration (FDA), as well as Good Laboratory Practice (GLP) standards.

S1.2 Summary of the analytical method

Tamsulosin concentrations in human sodium-heparin plasma were determined using a validated LC–MS/MS method with tamsulosin-d4 hydrochloride as the internal standard (IS). Sample preparation was performed using a liquid-liquid extraction (LLE) technique. Quantification was based on analyte-to-internal standard peak area ratios, and calibration curves were constructed by least-squares linear regression using a $1/x^2$ weighting factor.

S1.2.1 Linearity and calibration curve

Calibration standards were prepared in human plasma over the concentration range of 0.100-40.587 ng/mL. Calibration curves were constructed using analyte-to-internal standard peak area ratios across the studied concentration range.

The calibration model showed excellent linearity, with a mean correlation coefficient (r) of 0.99969.

Table S1. Measured concentrations and variability across three independent calibration runs for the evaluation of method linearity of tamsulosin in human plasma.

**Run**	**Nominal concentration** (ng/mL)							
	**0.100**	**0.200**	**0.998**	**4.329**	**8.658**	**17.317**	**31.658**	**40.587**
1	0.097	0.211	0.986	4.505	9.086	17.171	29.908	38.910
2	0.099	0.202	0.994	4.528	9.006	17.288	30.056	39.192
3	0.098	0.209	1.002	4.466	9.105	17.125	29.800	39.027
**Mean**	0.0980	0.2073	0.9940	4.4997	9.0657	17.1947	29.9213	39.0430
**SD**	0.00100	0.00473	0.00800	0.03134	0.05254	0.08404	0.12852	0.14168

**CV**	1.0	2.3	0.8	0.7	0.6	0.5	0.4	0.4

SD: standard deviation; %CV: coefficient of variation (%).

Table S2. Calibration curve parameters for tamsulosin.

Run	Slope (m)	Intercept (b)	Correlation coefficient (r)
1	0.9511	0.2632	0.9997
2	0.9577	0.2389	0.9997
3	0.9517	0.2496	0.9997
**Mean**	0.95351	0.25053	0.99969

Figure S1. Representative calibration curve obtained using analyte-to-internal standard peak area ratios, showing the linear relationship between concentration and response for tamsulosin in human plasma.

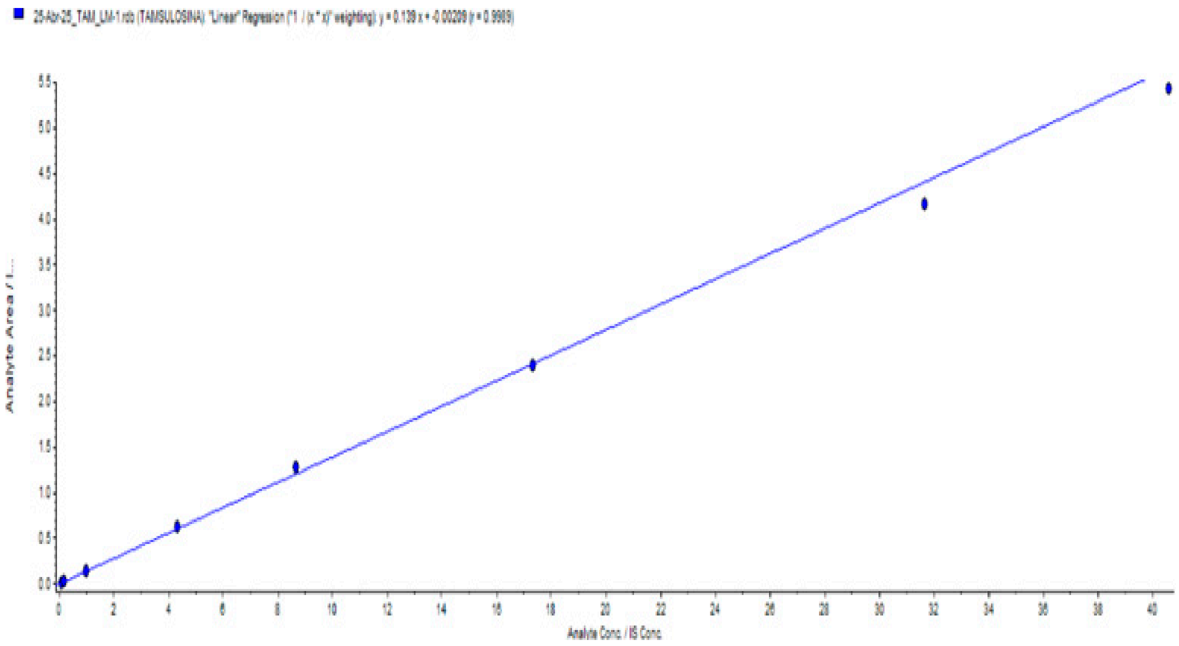

S1.2.2 Accuracy and precision

Intra-day and inter-day accuracy and precision were evaluated using quality control (QC) samples at multiple concentration levels.

For repeatability, six replicates of the lower limit of quantification (0.101 ng/mL), as well as QC samples at low, medium, and high concentrations (0.297, 13.394, and 31.149 ng/mL), were analyzed in sextuplicate. In addition, six replicates of the highest calibration standard diluted at ratios of 1/2 and 1/4 were evaluated.

For reproducibility, six replicates of the lower limit of quantification (0.101 ng/mL) and plasma quality control samples at low, medium, and high concentrations (0.297, 13.394, and 31.149 ng/mL) were analyzed in sextuplicate, with three determinations performed by each analyst on at least two different days.

The method showed adequate precision, with coefficients of variation (CV) below 15%, and accuracy expressed as absolute deviation (AD) within 15% of nominal concentrations across all QC levels. For the lower limit of quantification (LLOQ), both precision (CV ≤ 20%) and accuracy (AD ≤ 20%) were within the predefined acceptance criteria.

S1.2.3 Selectivity and matrix effect

Selectivity was evaluated using multiple individual plasma sources, including hemolyzed and lipemic samples, as well as plasma containing commonly co-administered drugs.

No significant interference was observed at the retention times of tamsulosin and the IS. Matrix effects were assessed and demonstrated acceptable variability, with normalized matrix factor CV values below 15% at both QC levels.

S1.2.4 Recovery

Extraction recovery was evaluated at different concentration levels. The method showed consistent recovery across the concentration range for both tamsulosin and the internal standard, with CV values below 15%. The mean relative recovery of tamsulosin was 94.99%.

S1.2.5 Lower limit of quantification (LLOQ)

The LLOQ was established at 0.100 ng/mL, demonstrating acceptable accuracy and precision (CV ≤ 20% and absolute deviation ≤20%), confirming the suitability of the method for quantifying low plasma concentrations.

S1.2.6 Stability

Stability of tamsulosin in plasma was evaluated at two concentration levels in human plasma analyzed in sextuplicate (0.297 and 31.149 ng/mL), under various conditions, including:

- Short-term (room temperature) stability (21.60 hours)
- Autosampler stability (10°C, 46.85 hours).
- Freeze–thaw cycles (6 cycles, -70°C).
- Long-term storage stability (-70°C, 155 days).
- Refrigerated processed sample stability (44.72 hours)
- Working solution stability (short- and long-term) (23.92 h and 155 days)
- Stock solution stability (short- and long-term) (23.83 h and 155 days)
- Whole blood stability. (3.90 hours)

Tamsulosin was found to be stable under all tested conditions, with precision (CV ≤ 15% and accuracy expressed as absolute deviation ≤ 15%) within the predefined acceptance criteria.

S1.3 Overall validation performance

The validation results demonstrated that the analytical method met all predefined acceptance criteria for bioanalytical method validation. The method showed adequate linearity, precision, accuracy expressed as absolute deviation, selectivity, recovery, sensitivity, and stability under all evaluated conditions.

Table S3. Summary of bioanalytical method validation results for tamsulosin.

Parameter	Acceptance criteria	Result
Linearity	r ≥ 0.98	0.99969
Precision (intra-day)	CV ≤ 15%	0.4-1.6%
Accuracy (intra-day)	Absolute deviation (AD) ≤15%	1.7- 5.2%
Precision (inter-day)	CV ≤ 15%	0.9-2.1%
Accuracy (inter-day)	AD ≤15%	2.1-6.7%

Selectivity	No interference at the retention time of the analyte and internal standard	No interference was observed at the retention time of tamsulosin and the IS in blank, hemolyzed, and lipemic plasma, or in the presence of tested drugs (salicylic acid acetaminophen, metamizole, ketorolac, ondansetron, loratadine, loperamide)
LLOQ (0.101 ng/mL)	CV ≤ 20%, AD ≤20%	CV 3.5%, AD 1.7
Carryover	Carryover at the analyte retention time < 20% and carryover at the internal standard (IS) retention time < 5%	0.0% for tamsulosin and 0.0% for tamsulosin-d4
Recovery	CV ≤ 15%	8.9% (tamsulosin), 5.6% (tamsulosin -d4)
Reinjection reproducibility	CV ≤ 15%, AD ≤ 15%	Stable up to 21.67 h (CV 0.5-2.0%, AD 1.7–2.9%)
Matrix effect	Normalized matrix factor CV ≤ 15% at both QC levels	1.3% (low QC), 0.5% (high QC)
Stability (short-term, room temperature)	CV ≤ 15%, AD ≤ 15%	Stable up to 21.60 (CV 1.0%-3.1%, AD 1.5%-2.4%)
Stability (autosampler at 10 °C)	CV ≤ 15%, AD ≤ 15%	Stable up to 46.85 h (CV 2.0%-2.2%, AD 0.9%-4.0%)
Freeze–thaw stability (-70°C, 6 cycles)	CV ≤ 15%, AD ≤ 15%	Stable for 6 cycles (CV 1.1%-1.7%, AD 0.2%-1.7%)
Long-term stability (-70°C, 155 days)	CV ≤ 15%, AD ≤ 15%	CV 0.4-1.7%, AD 9.3-10.00

Refrigerated processed sample stability (2-8°C)	CV ≤ 15%, AD ≤ 15%	Stable up to 44.72 h (CV 2.1-2.2%, AD 1.2-4.3%)
Working solution stability (short- and long-term)	AD ≤ 10%	Stable up to 23.92 h (high, short-term), 23.97 h (low, short-term), and 155 days (high and low,long-term)<br><br>CV 0.5–0.6% and AD 1.0-1.3% (high, short-term); CV 0.8-1.3% and AD 3.7-4.0% (low, short-term); CV 0.5-1.2% and AD 0.3–0.4% (high, long-term); CV 3.0-3.7% and AD 2.5-4.4% (low, long-term)
Stock solution stability (short- and long-term)	AD ≤ 10%	Stable up to 23.83 h (short-term) and 155 days (long-term); CV 0.3–0.5% and AD 4.9–5.6% (short-term); CV 0.4–0.7% and AD 1.2–3.3% (long-term)
Whole blood stability	CV ≤ 15%, AD ≤ 15%	Stable up to 3.90 h (CV 0.6–1.4%, AD 2.7–3.0%)
